# Ceftriaxone Resistance in Campylobacter Gastroenteritis

**DOI:** 10.7759/cureus.50632

**Published:** 2023-12-16

**Authors:** Megan D Hsu, An Phuc D Ta, Satori Iwamoto, Alexis Leo, Gary Chu

**Affiliations:** 1 College of Medicine, California Northstate University, Elk Grove, USA; 2 Internal Medicine, Kaiser South Sacramento, Sacramento, USA

**Keywords:** infectious diarrhea, meningitis-like symptoms, fluoroquinolone, ceftriaxone resistance, campylobacter enteritis

## Abstract

Annually, millions of people worldwide are exposed to *Campylobacter,* a species of bacteria that commonly causes gastroenteritis and in cases of immunocompromised individuals, can also lead to life-threatening complications. After stool cultures are obtained, the usual treatment for infectious diarrhea involves metronidazole and quinolones such as ciprofloxacin or levofloxacin. Quinolones are a family of broad-spectrum antibiotics known to be effective against various gram-negative infections that also include *Campylobacter jejuni (C. jejuni).* However, due to adverse side effects and bacterial resistance risks that may exist with medication use, they are no longer used as a first line. Our patient, initially treated with ceftriaxone for symptoms resembling bacterial meningitis, pneumonia, and infectious diarrhea, showed minimal to no improvement. Subsequent cerebral spinal fluid (CSF) ruled out meningitis while stool studies confirmed *C. jejuni *as the causative agent. A switch to levofloxacin resulted in a noticeable improvement in the patient’s condition. This case emphasizes the importance of considering changes in antibiotic regimen from ceftriaxone to quinolones when faced with persistent infectious diarrhea, due to the high prevalence of ceftriaxone resistance in *C. jejuni *infections.

## Introduction

*Campylobacter *affects approximately 1.5 million people annually in the United States and 2.4 million people worldwide [1]. It can be transmitted through untreated water and contact with farm animals [2]. Common symptoms include diarrhea, cramping, abdominal pain, fever, nausea, and vomiting, with potentially life-threatening complications in immunocompromised patients [3]. Diagnosis is confirmed through stool cultures. Quinolones such as levofloxacin and ciprofloxacin are accepted treatments for *Campylobacter* infections. Although deaths are not commonly reported, an estimated 24 deaths per 10,000 culture-confirmed cases highlight its severity [2].

Levofloxacin is a family of broad-spectrum antibiotics that is categorized in the (fluoro) quinolone group. It is used to treat various pulmonary infections including off-label use for those caused by the *Campylobacter *species [1]. Dosages range from 250-750 mg in IV and oral forms [4]. Its mechanism of action involves inhibiting the bacterial enzyme DNA-gyrase, also known as type II and type IV topoisomerase. This further prevents the relaxation of supercoiled DNA and promotes the breakage of double-stranded DNA [5]. Of note, this medication also carries a US black box warning for serious adverse reactions including tendinopathy, tendon ruptures, peripheral neuropathy, and CNS effects [5,6]. The potentially irreversible effect of tendon ruptures may pose a risk of career-ending injuries for athletes [7]. Additionally, individuals with myasthenia gravis may experience exacerbated muscle weakness due to the medication. Outside of the black box warning, other significant adverse reactions include increased risk of aortic aneurysm and dissection, arthropathy and arthralgias, *Clostridium difficile (C. diff)* infection, hyper and hypoglycemia, hepatotoxicity, photosensitivity/toxicity, QT-prolongation, and hypersensitivity reactions [8]. Given the severity of these adverse reactions, levofloxacin is reserved for use in patients who have no other alternative treatment options. 

Ceftriaxone, a third-generation cephalosporin antibiotic, is the recommended empiric treatment for community-acquired bacterial meningitis [9]. It is a broad-spectrum antibiotic that works by inhibiting bacterial wall synthesis by binding to penicillin-binding proteins. These proteins are enzymes necessary for peptidoglycan synthesis within the cell walls [10]. It is indicated for the treatment of bacterial meningitis caused by Haemophilus influenzae, Neisseria meningitidis, Streptococcus pneumoniae, Staphylococcus epidermidis, and Enterobacter coli but not the *Campylobacter* species [11]. Significant adverse reactions to consider encompass hypersensitivity reactions and increased susceptibility to *C. diff* infection. In particular for the pediatric population are considerations for ceftriaxone-calcium precipitation, immune hemolytic anemia, and Kernicterus arising from hyperbilirubinemia in neonates [12,13].

## Case presentation

A 48-year-old male with a past medical history of migraine, appendectomy, shoulder dislocation, and obstructive sleep apnea presented to the emergency department (ED) on August 19, 2023. He had symptoms of mid abdominal pain, nausea, hypotension, tachycardia, dark brown vomiting, nonbloody diarrhea, fevers with chills, and foul-smelling urine of dark color. Between August 11 and 13, the patient visited a lake where he swam and went on a boating ride. Additionally, he recently returned from a business dinner in Napa on August 17 where he visited a vineyard, tasted multiple wines, and experienced fine dining. No insect bites, bird-animal-toxic exposures, or illnesses from fellow travelers were noted. The patient had been relatively well until his symptoms began on August 18 around noon, starting with chills and severe body aches, followed by fever, nausea, emesis, and nonbilious, and non-bloody diarrhea. On August 19, he developed a headache and a purpuric rash around both ankles, feet, and abdomen. The patient attributed his illness to eating caviar for the first time during the business trip, which he reported to the ED.

During the physical examination, vital signs showed a blood pressure of 80/55 mmHg, heart rate of 139 bpm, respiratory rate of 18 breaths per min, oxygen saturation of 99% on room air, and a temperature of 98.9 ^o^F. The patient had dry lips, clear lungs, tachycardia without murmur, mid-abdomen tenderness on the left side without guarding, and suprapubic tenderness. Extremities were warm and dry, and the patient was alert and oriented to time, place, and location. 

**Figure 1:**
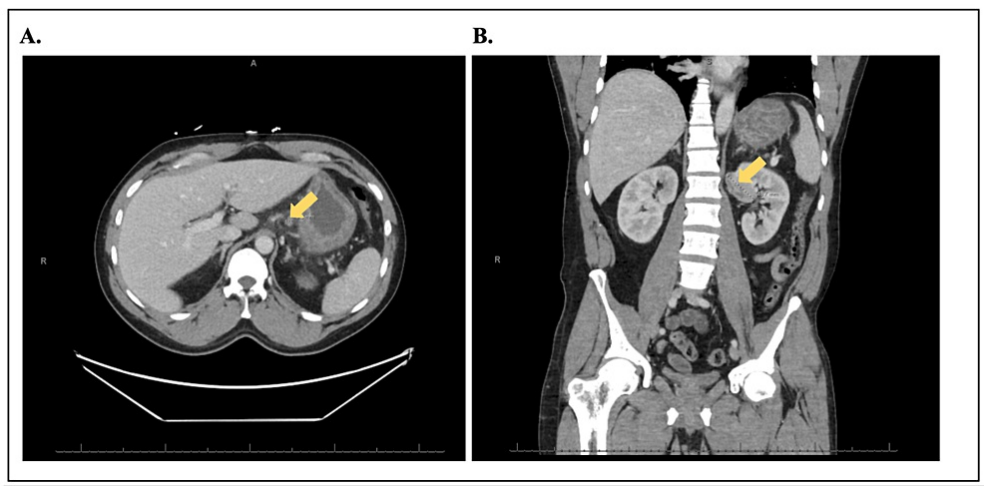
A. CT abdomen pelvis shows gastric mural edema with a diameter of 9.3 mm on day 1; B. CT abdomen pelvis shows the incidental finding of a left renal mass with a diameter of 48.7 mm on day 1

**Figure 2:**
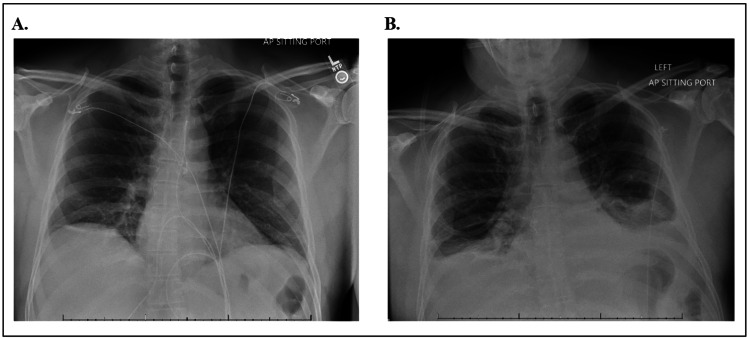
A. AP chest X-ray shows no pleural effusion on day 1 of hospitalization; B. AP chest X-ray shows mild to moderate pulmonary interstitial edema, suggesting pneumonia on day 4 of hospitalization

Laboratory workup was significant for leukocytosis, acute renal injury with a creatinine of 2.59 (Table 1), and metabolic acidosis with a lactate of 3.0. Radiologic images showed gastric mural edema (Figure 1A) and mild atelectatic changes involving the bilateral lower lobe, along with an incidental 4.9 cm mass near the upper pole of the left kidney per CT scan of the abdomen and pelvis (Figure 1B). The chest X-ray on day 1 was clear (Figure 2A). The diagnosis of sepsis was established, with the suspected causes being either meningitis or infectious diarrhea. A lumbar puncture was performed and stool samples were collected to rule out infectious diarrhea. The patient received IV fluids and was started on metronidazole (500 mg PO), vancomycin (1.25 g IV), ceftriaxone (2 g IV qd for 2 days), antiemetics (metoclopramide 10 mg IV prn), and analgesics. 

**Table 1:** Hospitalization lab values from admission on day 1 to discharge on day 11 The patient had hyponatremia on hospitalization days 2-4, 9, hypokalemia on day 6, hypercreatinemia days 1-8, renal insufficiency on day 1, elevated HGBA1C% on day 1, elevated ALT on day 7, elevated WBC days 2-10, elevated HCT days 3-11, and thrombocytopenia days 1, 3-8. eGFR: estimated glomerular filtration rate; HGBA1C%: hemoglobin A1C %; ALT: alanine transaminase; HCT: hematocrit; PLT: platelet

Day	1	2	3	4	5	6	7	8	9	10	11
Sodium (N: 135-145 mEq/L)	135	132	130	128		136	136	135	134	135	136
Potassium (N: 3.5-5.2 mEq/L)	4.2	4.6	4.2	3.7		3.1	3.5	3.9	3.9	3.8	4.1
Creatinine (N: 0.7-1.3 mg/dL)	2.59	2.77	3.82	2.72		1.72	1.41	1.42	1.31	1.3	1.3
eGFR (N ≥ 60 mL/min)	23										
Glucose (70-100 mg/dL)	70	99	124	103		97	97	87	85	90	89
HGBA1C (N < 5.7%)	5.8										
ALT (N: 7-55 U/L)	40	27					77			33	
WBC (N: 4.5-11 x 10^9^/L)	7.3	12.5	13.7	13.4	18.1	23	23.1	20.4	21.1	11.7	9.2
HGB (N: 14-18 g/dL)	14.5	13.7	12.8	12.1	12.3	13.3	13.6	12.5	12.8	12.6	13
HCT (N: 41-50%)	42.7	41	37.6	36.2	35.5	37.5	38.6	38.6	37.5	36.7	37
PLT (N: 150-350 x 10^9^/L)	136	143	102	70	70	101	107	132	182	211	252

**Figure 3:**
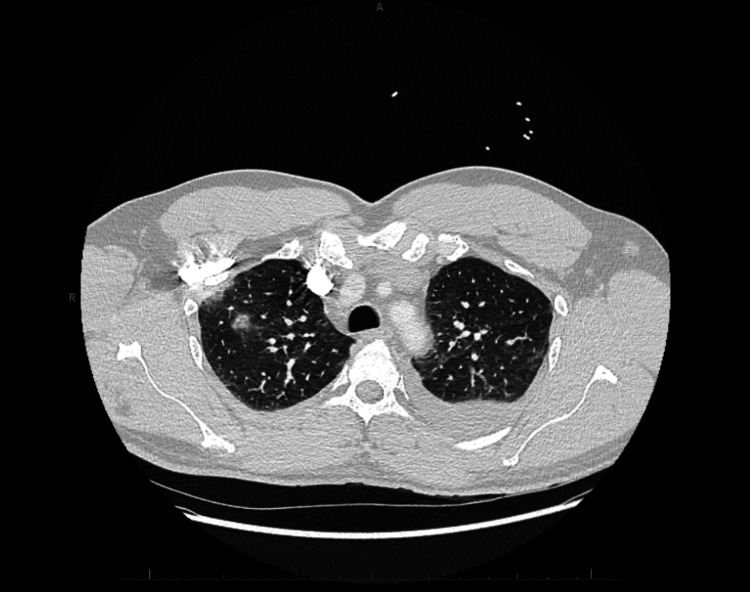
Axial CT of the chest shows bilateral pleural effusions with consolidations and ground-glass opacities, indicating pneumonia on day 6

Despite treating the renal injury with fluids and sepsis with broad-spectrum antibiotics on day 3, the patient showed minimal clinical improvement with good urine output. Follow-up chest X-ray revealed no infiltrate or pleural effusion. CSF studies, after 72 hours, returned negative for meningitis. Repeat chest X-ray and CT scan suggested pneumonia (Figures 2, 3). Antibiotics were changed to cefepime (2 g IV) on day 4 to see if the patient would have a better response. On day 7, the stool culture came back positive for *Campylobacter jejuni *(*C. jejuni*). Antibiotics were then changed to levofloxacin and doxycycline after consulting with an infectious disease specialist. The patient’s condition began to improve, and he was discharged home on hospital day 11 with oral levofloxacin and doxycycline. An outpatient urology referral was made for follow-up of the left renal mass, which was an incidental finding from the original CT scan.

## Discussion

*C. jejuni *is one of the leading causes of global bacterial gastroenteritis that, although uncommon, can present with other extraintestinal infections such as meningitis [14]. Interestingly, prior literature reports fewer than 10 cases of *C. jejuni* meningitis [15]. Meningitis caused by this gram-negative bacterium is extremely rare but can present as a severe infection of the meninges often seen in immunocompromised individuals. After initial infection of the gastrointestinal tract, *C. jejuni* can enter the bloodstream, leading to bacteremia and spread to other organs. While its exact mechanism of invasion remains unclear, *C. jejuni *can hematogenously spread beyond the gastrointestinal tract to the central nervous system and cause inflammation of the meninges [16]. However, in the case of our patient, their initial presentation of meningitis-like symptoms that included fever, chills, nausea, vomiting, headaches, and rash in the ankles, feet, and abdomen warranted preemptive treatment with antibiotics (ceftriaxone). Contrary to the patient’s meningitis-like symptoms, CSF test results came back negative for meningitis, but stool tests were positive for *C. jejuni*. While *Campylobacter* is typically transmitted through undercooked poultry, this case may provide unique insight into a potentially lesser-known association of *Campylobacter* with caviar, through untreated water [2,17]. Unpasteurized fish products, including caviar, are notorious for causing infectious diarrhea [17-19]. It may prove helpful to raise awareness of this more obscure association, especially in the Sacramento region of the United States, which is well known for its booming aquaculture industry and caviar market [20].

Meningitis-like symptoms generally require prompt medical attention and treatment to prevent additional complications that could arise with further progression of the infection [21]. Hospitalized patients often receive empirical treatment with third-generation broad-spectrum antibiotics, such as ceftriaxone, while awaiting bacterial culture results to identify the disease-causing pathogen or bacteria [22]. Culturing *C. jejuni* can take up to five days, as it is a thermophilic bacterium and requires approximately 48 to 72 hours to grow on a special growth medium [14,15]. In this case, the patient’s meningitis-like symptoms did not improve with ceftriaxone. However, upon stool culture results that came back positive for* C. jejuni*, the patient was switched to levofloxacin and doxycycline and showed rapid improvement afterward. While ceftriaxone is considered the first-line treatment for meningitis-like symptoms, it is not as effective in treating meningitis-like symptoms specifically caused by *C. jejuni*. In another literature review, it was found that of 122 patients infected with *C. jejuni,* only 1.6% were fully responsive to treatment with ceftriaxone, with the majority showing some level of resistance to the medication [23]. Additionally, although levofloxacin is not considered first-line for meningitis or general infections due to its side effects and increasing risk of resistance, it is used when there is a strong suspicion of Gram-negative bacteria such as *C. jejuni* [3]. Common adverse side effects of levofloxacin include but are not limited to arrhythmias from QT prolongation, diarrhea, lightheadedness, tendonitis, and headaches [24]. Furthermore, it should be noted that in many other parts of the world, *Campylobacter* is resistant to fluoroquinolones. In this case, macrolides would be the recommended treatment [25].

## Conclusions

Meningitis-like symptoms are an atypical presentation that, although rare, can be associated with *C. jejuni*. In the case of a patient who presents with bacterial meningitis and persistent diarrheal symptoms even after the administration of a broad-spectrum antibiotic, such as ceftriaxone, it is crucial to confirm the disease etiology with pertinent lab tests. While ceftriaxone is considered a first-line treatment for bacterial meningitis, additional culture studies may prove helpful when considering treatment with quinolones such as levofloxacin if symptoms persist post-ceftriaxone treatment.
